# Correction to: Climate change risk to pheromone application in pest management

**DOI:** 10.1007/s00114-021-01770-w

**Published:** 2021-12-15

**Authors:** Ashraf M. El‑Sayed, Suresh Ganji, Jürgen Gross, Natalie Giesen, Margit Rid, Peter L. Lo, Anna Kokeny, C. Rikard Unelius

**Affiliations:** 1 The New Zealand Institute for Plant and Food Research Limited, Gerald Street, Lincoln, 7608 New Zealand; 2 Department of Chemistry and Biomedical Sciences, Faculty of Health and Life Sciences, Linnaeus University, 392 31 Kalmar, Sweden; 3 Federal Research Centre for Cultivated Plants, Institute for Plant Protection in Fruit Crops and Viticulture, Julius Kuhn-Institut, Schwabenheimer Str. 101, 69221 Dossenheim, Germany; 4 The New Zealand Institute for Plant and Food Research Limited, Hawke’s Bay, Private Bag, 1401, Havelock North, 4157 New Zealand

**Correction to: The Science of Nature (2021) 108:47**

**https://doi.org/10.1007/s00114-021-01757-7**

In the original publication of this article contains error in the textbody.

1. The first sentence in the Abstract sections that reads: “Since of the first sex pheromone and the adoption of pheromone in pest management, the global pheromone market size has grown to reach USD 2.4 billion per year in 2019.” should have been “Since *the discovery* of the first sex pheromone and the adoption of pheromone in pest management, the global pheromone market size has grown to reach USD 2.4 billion per year in 2019.”

2. Figures 5 and 6 x-axes were changed “Temprature” to “Temperature”.

The original article has been corrected.

